# Effects of plyometric jump training on physical performance in female soccer players across the competitive level: a systematic review with meta-analysis of randomized controlled trials

**DOI:** 10.3389/fphys.2025.1675849

**Published:** 2025-10-01

**Authors:** Jordan Hernandez-Martinez, Bayron Coñapi-Union, Sebastian Canales-Canales, Joaquin Perez-Carcamo, Javier Sanchez-Sanchez, Mario Sánchez, Izham Cid-Calfucura, Edgar Vasquez-Carrasco, Tomás Herrera-Valenzuela, Braulio Henrique Magnani Branco, Pablo Valdés-Badilla

**Affiliations:** ^1^ Department of Physical Activity Sciences, Universidad de Los Lagos, Osorno, Chile; ^2^ Department of Education, Faculty of Humanities, Universidad de la Serena, La Serena, Chile; ^3^ Physical Education Pedagogy, Universidad de Los Lagos, Osorno, Chile; ^4^ Research Group Planning and Assessment of Training and Athletic Performance, Universidad Pontificia de Salamanca, Salamanca, Spain; ^5^ Department of Physical Activity, Sports and Health Sciences, Faculty of Medical Sciences, Universidad de Santiago de Chile (USACH), Santiago, Chile; ^6^ School of Occupational Therapy, Faculty of Psychology, Universidad de Talca, Talca, Chile; ^7^ Centro de Investigación en Ciencias Cognitivas, Faculty of Psychology, Universidad de Talca, Talca, Chile; ^8^ Graduate Program in Health Promotion, Cesumar University (UniCesumar), Maringá, Brazil; ^9^ Department of Physical Activity Sciences, Faculty of Education Sciences, Universidad Católica del Maule, Talca, Chile; ^10^ Sports Coach Career, Faculty of Life Sciences, Universidad Viña del Mar, Viña del Mar, Chile

**Keywords:** team sports, athletic performance, sports, plyometric training, muscle strength

## Abstract

This systematic review with meta-analysis aimed to update the analysis of the available body of peer-reviewed randomized controlled trials (RCTs) articles on the effect of plyometric jump training (PJT) on physical performance in female soccer players according to competitive level. Six databases (PubMed, Medline, Collection of Psychological and Behavioral Sciences, CINAHL Complete, Scopus and Web of Science) were searched. The PRISMA, TESTEX, RoB 2, and GRADE instruments were utilized to assess methodological quality and certainty of evidence. Effect sizes (Hedge’s g) for physical performance variables were calculated using both fixed and random-effects models. Subgroup analysis were conducted based on competitive level (amateur/professional), program duration, training frequency, and total number of sessions. The protocol was registered in PROSPERO (code: CRD42025634705). Fourteen RCTs were included, with 149 participants in 15 experimental groups and 139 in 14 control groups. Several meta-analysis were conducted, revealing significant improvements in countermovement jump (*p* = 0.07; ES = 0.59), squat jump (*p* = 0.00; ES = 0.76), drop jump (*p* = 0.01; ES = 1.65), maximum jump power (*p* = 0.00, ES = 0.85), Illinois test (*p* = 0.00, ES = 1.15), 20-m shuttle run test (*p* = 0.13, ES = 1.12), and ball kicking (*p* = 0.00; ES = 1.30). Subgroup studies revealed that PJT was more effective in professional soccer players than in amateur players (*p* = 0.03, ES = 0.54). Interventions with fewer than 16 sessions (*p* = 0.003, ES = 0.52), more than 6 weeks in duration (*p* = 0.05, ES = 1.42), and fewer than 2 sessions per week (*p* = 0.005, ES = 0.50) resulted in a significant improvement in countermovement jump. In conclusion, PJT significantly improved jump height, agility, speed lineal and kicking power tests in female soccer players.

**Systematic Review Registration:**

CRD42025634705.

## Introduction

Female participation in soccer has increased worldwide in recent years, reaching a total of 13.3 million female players in 2019, and is expected to reach 60 million by 2026 (Horan et al., 2023; Randell et al., 2021). This remarkable growth reflects not only a societal shift towards greater inclusivity in athletics but also an increasing recognition of the multifaceted benefits that soccer offers (Sanchez-Sanchez et al., 2025). Multiple actions are developed, such as running, turning, kicking and jumping (Martínez-Lagunas et al., 2014). Soccer-specific technical and tactical qualities, such as muscle strength, power, speed, endurance, and the ability to sprint repeatedly, determine a player’s success in the game (Martínez-Lagunas et al., 2014). Therefore, the design of effective training programs to improve the attributes of power, speed, repeated sprinting ability and endurance of soccer players is fundamental to optimize their performance during matches (Rodríguez-Fernández et al., 2024).

Plyometric jump training (PJT) consists of performing exercises that stimulate the stretch-shortening cycle, where the lengthening (eccentric) movement quickly follows a shortening (concentric) movement (Kons et al., 2023). This stretch-shortening cycle is related to the distribution of different mechanisms such as, for example, the accumulation of elastic energy, preload, increased muscle activation time, muscle history dependence (strength improvement), stretch reflexes, and muscle-tendon interactions (Kons et al., 2023) that facilitate greater mechanical work production in subsequent concentric muscle actions (Booth and Orr, 2016). It has become an alternative to traditional training that has demonstrated improvements in physical performance in female soccer players, such as in the height of vertical jumps (Ramirez-Campillo et al., 2020), linear sprinting, such as change-of-direction (COD) speed (Pardos-Mainer et al., 2021), and kicking performance (Sánchez et al., 2020). In a systematic review with a meta-analysis conducted by Ramirez-Campillo et al. (2020) in which eight randomized controlled trials (RCTs) were included, significant improvements in favor of PJT (ES = 1.01, *p* = 0.002) in countermovement jump (CMJ) compared with active control groups were reported, with no significant differences (*p* = 0.34 vs. 0.96) found by subgroup analyses (i.e., PJT frequency, duration and total number of sessions). Similar to the findings reported by Sánchez et al. (2020) in a meta-analysis of 13 RCTs in female soccer players, significant improvements in CMJ (ES = 0.71; *p* = 0.007), drop jump (DJ; ES = 0.79; *p* = 0.02), ball kicking (ES = 2.24; *p* = 0.03), linear sprint performance (ES = 0.78; *p* = 0.000) and COD speed (ES = 0.72; *p* = 0.000) in favor of PJT were reported in comparison with those reported in active control groups. No significant differences were found across the subgroups (i.e., PJT, frequency, duration and total number of sessions). Similarly, a meta-analysis with 12 RCTs performed by Pardos-Mainer et al. (2021) in female soccer players compared PJT vs. strength training, where only significant improvements in favor of PJT were reported in CMJ (ES = 2.42; *p* = 0.02), linear sprint performance (ES = 2.42; *p* = 0.02) and COD speed (ES = 2.99; *p* = 0.003) compared with active control groups. However, when meta-analysis by subgroup were performed (i.e., PJT frequency, duration and total number of sessions), no significant differences in favor of PJT compared with active control groups were reported in the three meta-analysis mentioned above (Pardos-Mainer et al., 2021; Ramirez-Campillo et al., 2020; Sánchez et al., 2020).

The evidence indicates that PJT is an effective training alternative to improve physical performance in decisive actions during soccer games, such as sprinting, jumping and kicking (Pardos-Mainer et al., 2021; Ramirez-Campillo et al., 2020; Sánchez et al., 2020), as well as a low-injury and safe method (Ramirez-Campillo et al., 2020). Given the growing scientific awareness of the relevance of PJT and the few reviews with meta-analysis of studies focused on female soccer players, it was considered appropriate to conduct a study of this type. To date, the effect of volume as a training dose is unknown, and the effect of PJT according to competitive level in female soccer players is not known. Therefore, this systematic review with meta-analysis aimed to update the analysis of the available body of peer-reviewed RCTs articles on the effect of PJT on physical performance in female soccer players according to competitive level.

## Methods

### Protocol and registration

The PRISMA guidelines were followed in this systematic review (Page et al., 2021). PROSPERO (the International Prospective Register of Systematic Reviews; ID code: CRD42025634705) has the protocol registered.

### Eligibility criteria

The inclusion criteria for this systematic review with meta-analysis were original and peer-reviewed articles published until July 2025 that were unrestricted by language or publication date. The materials excluded were conference abstracts, books and book chapters, editorials, letters to the editor, protocol records, reviews, case studies, and trials. In addition, this systematic review used the population, intervention, comparator, outcome, and study design (PICOS) framework (Liberati et al., 2009) (see Table 1).

**TABLE 1:** Selection criteria used in the systematic review with meta-analysis.

Category	Inclusion criteria	Exclusion criteria
Population	Apparently healthy female soccer players, with no restrictions as to their competition level or age	Female soccer players with health problems (e.g., injuries, recent surgery), and male soccer players
Intervention	A plyometric jump training program, defined as unilateral or bilateral lower body jumps, hops and lunges that typically use a pre-stretch or countermovement that emphasizes the stretch-shortening cycle (≥3 weeks 1 or more sessions per week)	Exercise interventions not involving plyometric jump training or exercise interventions involving plyometric jump training programs representing less than 50% of the total training load when delivered in conjunction with other training interventions (e.g., high-load resistance training)
Comparator	Active control group	Absence of active control group
Outcome	At least one physical performance measure of muscle power (i.e., jumping and/or ball kicking), linear and change of direction speed, or muscle strength before and after the training intervention	Lack of baseline and/or follow-up data
Study design	Randomized controlled trials	Non-randomized controlled trials

### Information search process and databases

The search process was conducted between May 2024 and July 2025 via six generic databases: PubMed, Medline, Sport Discus, CINAHL Complete, Scopus, and Web of Science (core collection). The search strategy: (“physical fitness” OR “physical performance” OR “conditional performance” OR “agility” OR “speed” OR “reaction time” OR “coordination” OR “balance” OR “explosive strength” OR “power” OR “endurance” OR “strength endurance”) AND (“jump” OR “plyometric jump” OR “plyometric” OR “plyometric exercise” OR “vertical jump” OR “countermovement jump” OR “drop jump” OR “reactive strength”) AND (“soccer” OR “football” OR “football soccer” OR “elite soccer” OR “professional soccer” OR “amateur soccer” OR “youth soccer” OR “college soccer” OR “semi-professional soccer”) AND (“female” OR “females” OR “women” OR “woman” OR “girl” OR “girls”). To assist in identifying additional relevant studies, two independent experts were consulted on the included publications and the inclusion and exclusion criteria. We stipulated two requirements for the experts: (i) to hold a PhD in sport science and (ii) to have peer-reviewed publications on physical performance in various population groups and/or physical performance published in journals with an impact factor according to Journal Citation Reports®. We did not disclose our search strategy to specialists to avoid bias in their searches. After completing these steps, we searched a database on 14 July 2025, for relevant retractions or errata related to the listed papers.

### Study selection and data collection process

The EndNote reference manager (version X9, Clarivate Analytics, Philadelphia, PA, United States) exported the studies. JHM and BCU conducted separate searches, eliminated duplicates, examined titles and abstracts, and examined complete texts. At this point, no disparities were discovered. The procedure was repeated for recommendations made by outside specialists and searches inside reference lists. The texts of possibly suitable papers were then examined, and the rationale behind excluding those not fitting the selection criteria was disclosed.

### Methodological quality assessment

TESTEX, a tool for exercise-based intervention studies (Smart et al., 2015), was used to assess the methodological quality of the chosen studies. One potential exclusion criterion was TESTEX results (Smart et al., 2015). According to Smart et al. (2015), there is a 15-point rating system (five points for study quality and 10 points for reporting). Two authors (JHM, BCU) carried out this process separately, whereas a third author (THV) served as a referee for cases that were borderline and needed further validation from another author (PVB).

### Data synthesis

The following data were obtained and analyzed from the selected studies: (i) author and year of publication; (ii) country of origin; (iii) study design; (iv) competitive level; (v) number of participants in the intervention and control group (CG); (vi), mean age of the sample; (vii) weight and height; (viii) activities performed in the PJT and CG; (ix) training volume (total duration, weekly frequency and time per session); (x) training intensity; (xi) physical performance assessments; (xii) number of jumps; (xiii) intensity; (xiv) surface types; and (xv) experience in plyometric improvement.

### Risk of bias in individual studies

Two independent researchers (JHM and ICC) evaluated the risk of bias version 2 (RoB 2) of the included studies, and a third researcher (PVB) analyzed the results. The Cochrane Handbook for Systematic Reviews of Interventions’ recommendations for RCTs was the foundation for this evaluation (Sterne et al., 2019). On the basis of the randomization procedure, departures from the planned interventions, missing outcome data, outcome assessment, and choice of the reported result, the risk of bias was categorized as “high”, “low”, or “some concerns”.

### Summary measures for meta-analysis

The study methodology includes meta-analysis; complete information is accessible at PROSPERO (registration code: CRD42025634705). Meta-analysis were only performed in the present case when ≥3 studies were available (Ramirez-Campillo et al., 2022). Effect sizes (ES; Hedge’s g) for each jump performance, COD speed, sprint performance, and ball kicking performance in the PJT and CG were calculated via the pretraining and post training means and SD (standard deviations) for each dependent variable. The data were standardized according to the change score (SD). The ES values are presented with 95% confidence intervals (95% CIs). The calculated ES was interpreted via the following scale: trivial: <0.2; small: 0.2–0.6; moderate: >0.6–1.2; large: >1.2–2.0; very large: >2.0–4.0; extremely large: >4.0 (Hopkins et al., 2009). The random effects model was used to account for differences between studies that might affect the effect of PJT. Comprehensive meta-analysis software (Version 2.0; Biostat, Englewood, NJ, United States) was used. Statistical significance was set at *p* ≤ 0.05 (Verhagen et al., 1998) and was used to perform these calculations. In each trial, the random effects model (Der Simonian‒Laird approach) was used to calculate and pool the standardized mean difference (SMD) and mean difference (MD) of CMJ, SJ, DJ, peak jump power, Illinois test, 20-m sprint, and ball kicking dominant and non-dominant foots (PJT vs. CG). The fundamental premise of the random-effects model is that genuine effects (interventions, duration, among others) vary throughout studies and that samples are selected from populations with varying ES. The data were pooled if at least three studies presented the same results (Davey et al., 2011).

Heterogeneity between trial results was tested with a Cochran’s Q test (Morris et al., 2008) and the I^2^ statistic. I^2^ values of <25%, 25%–50%, and >50% represent small, medium, and large amounts of inconsistency, respectively (Higgins et al., 2003). Egger regression tests were performed to detect small study effects and possible publication bias (Higgins and Thompson, 2002).

### Moderator analyses

Using a random effects model and independent computed single factor analysis, potential sources of heterogeneity likely to influence the effects of training were selected *a priori*.

### Subgroup analyses

As adaptive responses to PJT programs may be affected by participants’ competitive level (Toselli et al., 2022), these factors were considered potential moderator variables.

### Single training factor analysis

Single training factor analyses were computed for the program duration (number of weeks and total number of training sessions) (de Villarreal et al., 2009) and training frequency (number of sessions per week) (Sáez-Sáez de Villarreal et al., 2010) on the basis of the reported influence of these variables on physical performance adaptations to PJT.

### Meta-regression

A multivariate random-effects meta-regression was conducted to verify whether any of the training variables (frequency, duration, and total number of sessions) predicted the effects of PJT on the physical performance variables. The meta-regression was computed with at least 10 studies per covariate (Higgins and Green, 2008).

### Certainty of evidence

Studies were categorized as having high, moderate, low, or very low confidence on the basis of their assessment of the GRADE scale (Guyatt et al., 2011). Because studies with RCT designs were included, all analyses began with a high degree of certainty and were downgraded if there were concerns about bias, consistency, accuracy, precision, directness of results, or risk of publication bias (Guyatt et al., 2011). Two authors evaluated the studies separately (JHM, BCU), and any disagreements were settled by agreement with a third author (PVB).

## Results

### Study selection

**FIGURE 1:**
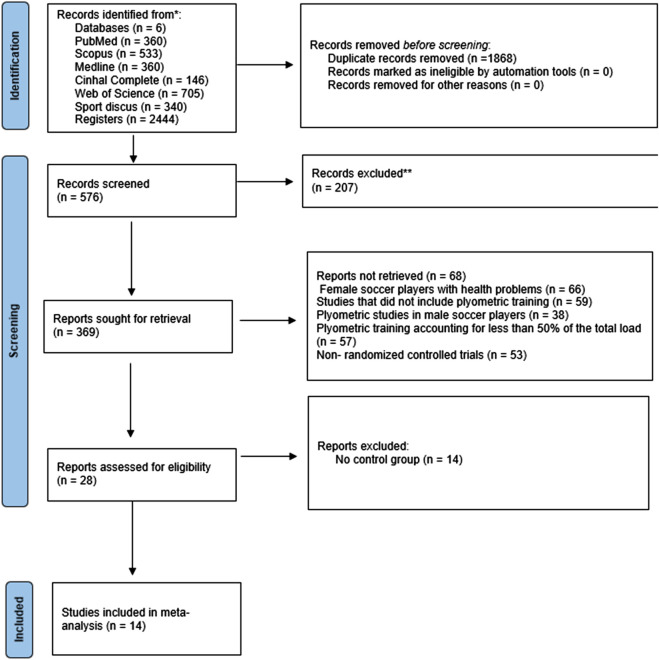
Flowchart of the review process. Legends: Based on the PRISMA guidelines (Page et al., 2021).

Figure 1 details the search process for the studies. A total of 2,444 records were found. Subsequently, duplicates were eliminated, and the studies were filtered by selecting the title, abstract, and keywords, resulting in 576 references. In the subsequent analysis phase, 207 articles were excluded because the texts did not meet the search criteria, leaving 369. Subsequently, 66 female soccer players with health problems, 59 studies that did not include plyometric training, 38 plyometric studies in male soccer players, 57 plyometric training accounting for less than 50% of the total load, and 53 non-randomized controlled trials. After this process, 28 potential studies remained, of which 14 were excluded case studies. Therefore, 14 studies met all selection criteria (Fischetti et al., 2019; Leon Muñoz et al., 2024; Maciejczyk et al., 2021; Morris et al., 2008; Nonnato et al., 2022; Ozbar et al., 2014; Porrati-Paladino and Cuesta-Barriuso, 2021; Ramirez-Campillo et al., 2018a; Ramírez-Campillo et al., 2016; Rosas et al., 2017; Rubley et al., 2011; Sanchez et al., 2022; Sedano Campo et al., 2009). The included studies involved 149 participants in 15 experimental groups and 139 participants in 14 control groups. The characteristics of the participants and the PJT interventions used in the included studies are displayed in Table 2.

**TABLE 2:** Characteristics of participants examined in the included studies.

Author	RCT	N/Country		Competitive level	Years	Body weight (kg)	Height (cm)	Training			Number of jumps	Intensity	Surface types	Experience in plyometric improvement
								Weeks	Frequency	Minutes				
Porrati-Paladino and Cuesta-Barriuso (2021)	Yes	PJT:8 CG:7	Spain	University	PJT:21.11 ± 4.16 CG: 22.38 ± 2.8	PJT: 62.38 ± 4.34 CG: 66.16 ± 15.81	PJT: 163 ± 4.63 CG:162.38 ± 4.34	6	3	20 12	70	NR	NR	NR
Ramirez-Campillo et al. (2018b)	Yes	PJT1:8 PJT2:8 CG:7	Chile	Amateur	PJT1: 22.8 ± 4.3 PJT2: 21.4 ± 2.5 CG: 20.1 ± 1.8	PJT1: 54.9 ± 3.7 PJT2: 59.6 ± 8.5 CG: 55.3 ± 3.3	PJT1: 158.0 ± 3.0 PJT2: 157.6 ± 4.8 CG: 160.1 ± 5.0	8	2	120	255–510	NR	natural grass	≥2 years of systematic training and competitive experience
Rosas et al. (2017)	Yes	PJT:8 CG:8	NR	Amateur	PJT: 22.8 ± 2.1 CG: 24.0 ± 2.7	PJT: 61.1 ± 8.3 CG: 58.5 ± 7.2	PJT: 1.64 ± 0.08 CG: 1.62 ± 0.04	6	2	120	140–260	NR	NR	NR
Leon Muñoz et al. (2024)	Yes	PJT:11 CG:12	Spain	Professional	PJT: 23.4 ± 3.96 CG: 19.9 ± 1.73	PJT: 57.3 ± 3.8 CG: 60.3 ± 7.55	PJT: 163 ± 3.41 CG: 163 ± 7.75	6	2	20		NR	NR	NR
Fischetti et al. (2019)	Yes	PJT:14 CG:14	Italy	Professional	PJT: 26 ± 6.9 CG: 26 ± 5.3	PJT: 60.6 ± 4.6 CG: 60.8 ± 6.3	PJT: 160.7 ± 4.1 CG: 160.1 ± 4.3	12	3	40–60	3,420	NR	Synthetic hard floor	≥5 years of soccer training and at least 3 years of plyometric training
Sedano Campo et al. (2009)	Yes	PJT:10 CG:10	Spain	Elite	PJT: 22.8 ± 2.1 CG: 23.0 ± 3.2	PJT: 58.7 ± 3.1 CG: 56.1 ± 2.6	PJT: 163.0 ± 7.0 CG: 161.5 ± 5.4	12	3	40–60	3,320	NR	Hard synthetic flooring	5.2 ± 3.2 years Soccer experience
Sanchez et al. (2022)	Yes	PJT:8 CG:6	NR	Professional	PJT: 16.0 ± 2.3 CG:16.0 ± 2.7	PJT: 60.7 ± 6.3 CG:56.5 ± 12.8	PJT: 161.9 ± 6.2 CG:164.0 ± 6.8	4	NR	20	320	RPE 5 to 8	NR	≥12 months of systematic soccer and strength training
Maciejczyk et al. (2021)	Yes	PJT:7 CG:8	Poland	Professional	PJT: 21 ± 3 CG:18.2 ± 1.8	PJT: 61.3 ± 13.86 CG:55.0 ± 5.39	PJT: 164.5 ± 6.91 CG: 161.7 ± 4.3	3	2	NR	524	NR	NR	9.75 ± 3.75 and 8.9 ± 2.5 Years of experience, respectively
Morris et al. (2008)	Yes	PJT: 11 CG:11	Argelia	Professional	PJT: 15.08 ± 0.19 CG:15.16 ± 0.93	PJT: 50.33 ± 6.11 CG:49.66 ± 6.02	PJT: 153.00 ± 6.47 CG:151.5 ± 5.57	10	3	15	900	NR	NR	≥4 ± 0.53 years of experience
Nonnato et al. (2022)	Yes	PJT:8 CG:8	NR	Professional	NR	Both groups: 60.3 ± 4.9	Both groups 1.67 ± 3.7	12	4	80	6,720	NR	NR	NR
Ramírez-Campillo et al. (2016)	Yes	PJT:10 CG:10	Chile	Amateur	PJT: 22.9 ± 1.7 CG: 22.5 ± 2.1	PJT: 56.8 ± 5.4 CG: 60.1 ± 7.5	PJT: 1.64 ± 0.09 CON: 1.61 ± 0.06	6	2	120	NR	NR	NR	NR
Rubley et al. (2011)	Yes	PJT:10 CG:6	NR	University	NR	Both groups:50.84 ± 5.1	Both groups: 162.5 ± 5.67	14	3	80	528	NR	NR	>4 years of soccer experience
Ozbar et al. (2014)	Yes	PJT:9 CG:9	Turkey	University	PJT:18.3 ± 2.6 CG:18.0 ± 2.0	PJT: 58.8 ± 7.8 CG: 54.4 6 6.1	PG: 163.1 ± 5.3 CON: 159.4 ± 5.1	8	1	60	1,210	NR	Pasto natural	≥4 Years of Training
Ramírez-Campillo et al. (2016)	Yes	PJT:19 CG:19	Chile	University	PJT:22.4 ± 2.4 CG:20.5 ± 2.5	PJT:60.7 ± 9.3 CG:60.2 ± 9.3	FPT:161 ± 5 FCG:159 ± 6	6	2	120	1440	NR	NR	more than 2 years of systematic soccer training and compe-titive experience

RCT: randomized controlled trial; PJT: plyometric jump training; CG: control group; NR: not reported.

### Methodological quality

The 14 selected studies were analyzed via the TESTEX scale (Table 3). All the studies achieved a score equal to or greater than 60% on the TESTEX scale (Fischetti et al., 2019; Leon Muñoz et al., 2024; Maciejczyk et al., 2021; Morris et al., 2008; Nonnato et al., 2022; Ozbar et al., 2014; Porrati-Paladino and Cuesta-Barriuso, 2021; Ramirez-Campillo et al., 2018a; Ramírez-Campillo et al., 2016; Rosas et al., 2017; Rubley et al., 2011; Sanchez et al., 2022; Sedano Campo et al., 2009), namely, 9/15 (Fischetti et al., 2019; Leon Muñoz et al., 2024; Rubley et al., 2011; Sedano Campo et al., 2009), 10/15 (Maciejczyk et al., 2021; Morris et al., 2008; Nonnato et al., 2022; Ozbar et al., 2014), 11/15 (Ramírez-Campillo et al., 2016; Sanchez et al., 2022), 12/15 (Ramirez-Campillo et al., 2018b; Ramírez-Campillo et al., 2016; Rosas et al., 2017), and 13/15 (Porrati-Paladino and Cuesta-Barriuso, 2021).

**TABLE 3:** Study quality assessment according to the TESTEX scale.

Study	Eligibility Criteria specified	Randomly Allocated Participants	Allocation Concealed	Groups similar at baseline	Assessors Blinded	Outcome Measures assessed >85% of participants*	Intention to treat analysis	Reporting of between group statistical comparisons	Point measures and measures of variability reported **	Activity monitoring in control group	Relative exercise Intensity reviewed	Exercise volume and energy expended	Overall TESTEX#
Nonnato et al. (2022)	Yes	Yes	Yes	Yes	No	Yes (1)	Yes	Yes	Yes (1)	Yes	No	Yes	10/15
Sedano Campo et al. (2009)	Yes	Yes	No	Yes	No	Yes (1)	No	Yes	Yes (2)	Yes	No	Yes	9/15
Leon Muñoz et al. (2024)	Yes	Yes	No	Yes	No	Yes (1)	No	Yes	Yes (2)	Yes	No	Yes	9/15
Fischetti et al. (2019)	Yes	Yes	No	Yes	No	Yes (1)	No	Yes	Yes (2)	Yes	No	Yes	9/15
Maciejczyk et al. (2021)	Yes	Yes	No	Yes	No	Yes (1)	No	Yes	Yes (2)	Yes	Yes	Yes	10/15
Sanchez et al. (2022)	Yes	Yes	Yes	Yes	Yes	Yes (1)	No	Yes	Yes (2)	Yes	Yes	Yes	12/15
Morris et al. (2008)	Yes	Yes	No	Yes	No	Yes (1)	No	Yes	Yes (2)	Yes	Yes	Yes	10/15
Ozbar et al. (2014)	Yes	Yes	No	Yes	No	Yes (1)	No	Yes	Yes (2)	Yes	Yes	Yes	10/15
Porrati-Paladino and Cuesta-Barriuso (2021)	Yes	Yes	Yes	Yes	Yes	Yes (1)	Yes	Yes	Yes (2)	Yes	Yes	Yes	13/15
Ramirez-Campillo et al. (2018a)	Yes	Yes	Yes	Yes	Yes	Yes (1)	No	Yes	Yes (2)	Yes	Yes	Yes	12/15
Ramírez-Campillo et al. (2016)	Yes	Yes	Yes	Yes	Yes	Yes (1)	No	Yes	Yes (2)	Yes	Yes	Yes	12/15
Rubley et al. (2011)	Yes	Yes	No	Yes	No	Yes (1)	No	Yes	Yes (1)	Yes	Yes	Yes	9/15
Rosas et al. (2017)	Yes	Yes	Yes	Yes	Yes	Yes (1)	No	Yes	Yes (2)	Yes	Yes	Yes	12/15
Ramírez-Campillo et al. (2016)	Yes	Yes	Yes	Yes	Yes	Yes (1)	No	Yes	Yes (2)	Yes	Yes	Yes	12/15

*Three points are possible: one point if adherence >85%, one point if adverse events were reported, and one point if exercise attendance was reported. **Two points possible: one point if the primary outcome is reported and one point if all other outcomes are reported. # total out of 15 points. TESTEX: tool for assessing study quality and reporting in exercise.

### Risk of bias

**FIGURE 2:**
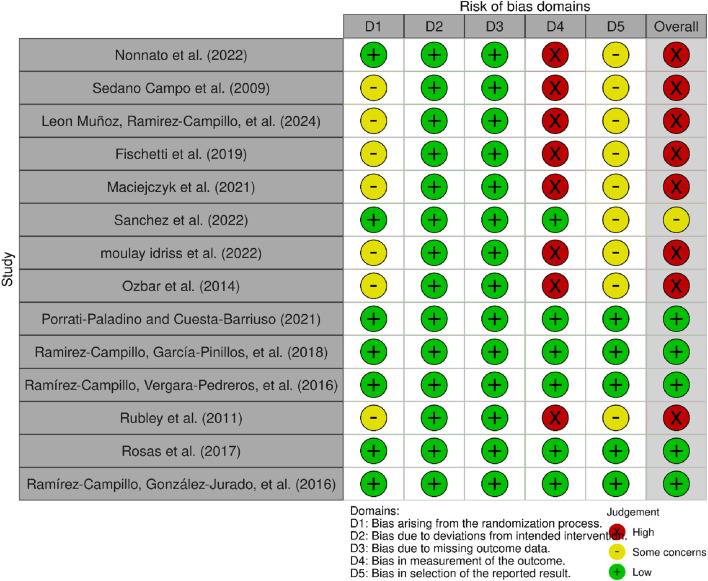
Risk of bias within studies. Legends: D1: randomization process; D2: deviations from the intended interventions; D3: missing outcome data; D4: measurement of the outcome; D5: selection of the reported result.

**FIGURE 3:**
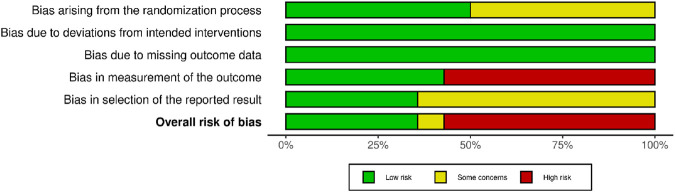
Risk of bias summary: Review the authors; judgments about each risk of bias item in each included study.

The risk of bias was low in 5 studies (Porrati-Paladino and Cuesta-Barriuso, 2021; Ramirez-Campillo et al., 2018a; Ramírez-Campillo et al., 2016; Rosas et al., 2017), low in 1 study (Sanchez et al., 2022), and high in 8 studies (Fischetti et al., 2019;Leon Muñoz et al., 2024; Maciejczyk et al., 2021; Morris et al., 2008; Nonnato et al., 2022; Ozbar et al., 2014; Rubley et al., 2011; Sedano Campo et al., 2009). All results for each domain: (i) randomization process; (ii) deviations from planned interventions; (iii) missing outcome data; (iv) outcome measurement; (v) selection of the reported outcome. These results are presented in Figures 2, 3.

### Meta-analysis results

The overall effects of PJT on the physical performance variables are shown in Table 4. The forest plots are shown in Supplementary Figures S1–S12. There were significant large effects (*p* < 0.05) in favor of PJT in 20-m speed, SJ, DJ, peak power jump, Illinois test, 20-m shuttle run test and ball kicking (ES = 0.62–1.30). However, at the CMJ, there were no significant differences (*p* > 0.05) with small to large effect sizes (ES = 0.01–1.12).

**TABLE 4:** Synthesis of the results of the included studies on the effects of plyometric jump training in physical performance in female soccer players.

	n^a^	ES (95%CI)	*Model of effect*	*p*	*I* ^2^ (%)	Egger’s test (p)	RW (%)
Jump performance							
CMJ (cm)	12. 13. 12. 266	0.59 (−0.06–1.24)	Random	0.07	83.2	0.00	5.33 to 9.03
SJ (cm)	5.5.5. 89	0.76 (0.34–1.17)	Random	**0.00**	71.9	0.01	21.7 to 26
DJ (cm)	5.6.5. 118	1.65 (0.69–2.60)	Random	**0.01**	78.9	0.00	3.03 to 6.28
Peak power jump (w)	3.3.3. 54	0.85 (0.32–1.38)	Fixed	**0.00**	**0.00**	**0.47**	11 to 12.3
Agility							
Illinois (sec)	4.4.4. 90	1.15 (0.74–1.56)	Random	**0.00**	71.3	0.01	20.1 to 22
Sprint performance							
20-m speed (sec)	4.4.4. 71	0.62 (0.17–1.07)	Random	**0.01**	87.4	0.00	11.4 to 17.7
20-m shuttle run test (sec)	3.3.3. 75	1.12 (−0.34–2.59)	Random	0.13	87.4	0.00	0.78 to 5.34
Kicking performance							
Ball kicking (km/h) (m)	3.4.3. 60	1.30 (0.71–1.89)	Fixed	**0.00**	0.00	0.00	11.0 to 21.0

Bolded p values indicate significant improvement (*p* < 0.05) in the experimental group after plyometric training compared with the control group, and *p* > 0.05 represents a low risk of publication bias. CMJ, Counter movement jump; SJ, Squat jump; DJ, Drop jump.

^a^ Data indicate the number of studies that provided data for analysis, the number of experimental and control groups, and the total number of female soccer players included in the analysis. Abbreviations: 95% CI, 95% confidence interval; ES, effect size (Hedge’s g); RW, relative weight of each study in the analysis.

### Meta-analysis subgroup

#### Subgroup analysis by competitive level

There were no significant differences in CMJ among female professional soccer players (6 experimental groups; *p* = 0.08; ES = 0.43; 95% CI = −0.28–1.15; I^2^ within the group = 73.9%). On the other hand, significant differences in CMJ were found among female amateur soccer players (4 experimental groups; *p* = 0.03; ES = 0.54; 95% CI = 0.04–1.04; I^2^ within the group = 0.00%).

#### Subgroup analysis by total number of sessions

There were no significant differences in CMJ across more than 16 sessions (5 experimental groups; *p* = 0.29; ES = 1.06; 95% CI = −0.60–2.73; I^2^ within the group = 93.5%). CMJ was significantly different across the 8 experimental groups (*p* = 0.003; ES = 0.52; 95% CI = 0.18–0.86; I^2^ within the group = 0.00) in <16 total sessions.

#### Subgroup analysis by training duration

CMJ did not significantly differ after less than 6 weeks of training (7 experimental groups; *p* = 0.60; ES = 0.14; CI95% = −0.41–0.71; I^2^ within the group = 65.9). In contrast, CMJ (6 experimental groups; *p* = 0.05; ES = 1.42; CI95% = −0.01–2.85; I^2^ within the group = 90.1) was significantly different after >6 weeks of training.

#### Subgroup analysis by frequency of training

No significant differences were observed in CMJ across >2 weekly sessions (6 experimental groups: *p* = 0.19; ES = 0.95; CI95% = −0.47–2.37; I^2^ within the group = 92.1). The number of CMJ was significantly different among the 7 experimental groups (*p* = 0.005; ES = 0.50; CI95% = 0.15–0.86; I^2^ within group = 0.00) in <2 sessions per week.

## Results of the meta-regression

The calculation of the meta-regression was performed with at least 10 studies per covariate. Only CMJ was considered for the meta-regression analysis, which analyzed 3 training variables (frequency, duration and total number of sessions) (Table 5). Regardless of training type, none of the training variables were found to predict the effects of PJT on CMJ performance (*p* > 0.05).

**TABLE 5:** Results of the multivariate random-effect meta-regression for training variables to predict plyometric jump training effects on countermovement jump performance in female soccer players.

Covariate	Coefficient	95% Cl	Z	P	*R* ^2^
Countermovement jump height (n = 13)					
Intercept	0.50	−1.90 to 2.92	0.41	0.67	0.00
Duration	−0.07	0.38 to 0.23	−0.49	0.62	0.00
Frequency	−0.21	−0.88 to 0.44	−0.65	0.51	0.00
Total, sessions	0.05	−0.03 to 0.14	1.19	0.23	0.00

n indicates the number of study groups. Bolded p values indicate a significant (p < 0.05) prediction effect of plyometric jump training on jumping performance.

^a^ Computation of meta-regression was performed with at least 10 studies per covariate, available only for countermovement performance from the investigated fitness variables. Abbreviations: 95% CI, 95% confidence interval.

### Certainty of evidence

The results of the certainty of evidence range from low to high, which only allows recommendations to be made for agility and ball kicking performance on the use of PJT interventions on physical performance variables concerning CG in female soccer players (Table 6).

**TABLE 6:** GRADE assessment for the certainty of evidence.

Assessment of certainty							Number of patients		Effect		Certainty	Importance
Number of studies	Study design	Risk of bias	Inconsistency	Indirect evidence	Vagueness	Other considerations	Plyometric jump training	Active controls	Relative (95% CI)	Absolute (95% CI)		
Vertical jump												
12	randomized trials	very serious ^to^	It is not serious	It is not serious	It is not serious	none	141/276 (51.1%)	135/276 (48.9%)	not estimable		⨁⨁ ◯◯ Go down ^to^	IMPORTANT
Agility												
4	randomized trials	It is not serious	It is not serious	It is not serious	It is not serious	none	35/71 (49.3%)	36/71 (50.7%)	not estimable		⨁⨁⨁⨁ High	IMPORTANT
Ball kicking performance												
4	randomized trials	serious^b^	It is not serious	It is not serious	It is not serious	none	34/53 (64.2%)	19/53 (35.8%)	not estimable		⨁⨁⨁ ◯ Moderate^b^	IMPORTANT
Sprint performance												
4	randomized trials	very serious ^to^	It is not serious	It is not serious	It is not serious	none	36/71 (50.7%)	35/71 (49.3%)	not estimable		⨁⨁ ◯◯ Go down ^to^	IMPORTANT

## Discussion

This systematic review with meta-analysis aimed to update the analysis of the available body of peer-reviewed RCTs articles on the effect of PJT on physical performance in female soccer players according to competitive level. Our findings revealed that PJT is effective in improving jump height in CMJ, SJ, and DJ, peak vertical jump power, kicking performance, and time in 20-m speed and Illinois tests in female soccer players. On the other hand, our subgroup meta-analysis by competitive level revealed significant differences in CMJ among female professional soccer players. Subgroup analysis by total number of sessions revealed significant differences for CMJ in <16 total sessions, whereas subgroup analysis by training duration revealed significant differences in CMJ with training durations of >6 weeks. Finally, subgroup analysis by training frequency revealed significant differences for CMJ with frequencies of <2 sessions per week.

### Countermovement jump

Our meta-analysis revealed no significant improvement in CMJ in favor of PJT compared with the control conditions (ES = 0.59; *p* = 0.07). However, Sánchez et al. (2020) in a meta-analysis of 13 RCTs of PJT in female soccer players, reported significant improvements in CMJ (ES = 0.71; *p* = 0.007). This finding is in line with that reported by Ramirez-Campillo et al. (2020) in a meta-analysis with 8 RCTs of PJT in female soccer players, who reported significant increases in CMJ (ES = 1.01; *p* = 0.002). Similarly, Pardos-Mainer et al. (2021), in a meta-analysis with 12 RCTs in female soccer players, compared PJT with strength training and reported significant improvements in favor of PJT in CMJ (ES = 2.42; *p* = 0.02). Additionally, Stojanović et al. (2017), in a meta-analysis with six studies of PJT in female soccer players, reported a moderate effect in favor of CMJ (ES = 1.09). Although our finding is unusual, it may be attributed to the high level of heterogeneity observed in our meta-analysis (I^2^ = 83.2%), which reflects substantial variability among studies regarding intervention duration, frequency, intensity, and exercise selection—factors that may have influenced the overall effect on CMJ performance. Moreover, the small sample size of some studies may have limited the statistical power. It is well established that PJT can enhance CMJ performance through improvements in neuronal activation patterns and optimization of the stretch-shortening cycle (SSC) following training (Komi and Gollhofer, 1997; Stojanović et al., 2017). Specifically, PJT can stimulate the recruitment of high-threshold motor units and induce adaptations in neuromuscular activity, muscle fiber size and length, as well as myotendinous junction stiffness, thereby improving force application during the jump (Duchateau and Amiridis, 2023; Suchomel et al., 2019). The utilization of elastic energy also depends on the coupling time between the pre-stretch and subsequent shortening phases, since a longer interval between eccentric and concentric phases increases the proportion of elastic energy lost and dissipated as heat (Duchateau and Amiridis, 2023). In this regard, although the CMJ is considered a slow SSC (>250 ms) (Nicol et al., 2006), PJT has been shown to be beneficial for muscle function during this task.

### Squat jump

Our meta-analysis reported significant effects in favor of PJT on SJ (ES = 0.76; *p* = 0.000). Unlike the findings of Stojanović et al. (2017) in a meta-analysis with only two studies of PJT in female soccer players, who reported a small effect on SJ (ES = 0.44; 95% CI -0.09–0.97). In our meta-analysis, only one study reported no significant improvements in SJ (ES = 0.06; *p* = 0.889), although this could be attributed to the low training frequency used, since they performed only one session per week of PJT for 12 weeks, which could be an insufficient stimulus to improve SJ in professional female soccer players (Nonnato et al., 2022). In contrast, Maciejczyk et al. (2021), who performed a PJT intervention with a frequency of two sessions per week for 4 weeks in professional female soccer players, reported significant improvements in SJ (*p* = 0.01). Similarly, Morris et al. (2008) reported significant improvements in SJ (*p* < 0.05) after 10 weeks of PJT training, with a frequency of three times per week in adolescent female soccer players. Importantly, few intervention studies with PJT have assessed SJ, which could be attributed to the fact that SJ is characterized by being a concentric jump only because the three to 5 seconds rest in a half-square position before its execution; thus, during the amortization phase, the elastic potential energy dissipates, which reduces the positive effects on the SSC. In this sense, SJ is considered a ballistic and not a plyometric exercise, so its application as a test during PJT interventions may lack specificity (Ünver et al., 2024). Our findings suggest that PJT may have improved lower limb concentric strength, specifically of the extensor muscles, during the jump (Kons et al., 2023). PJT can generate physiological adaptations, including increased muscle fiber strength, power capacity, and electromyographic activity (e.g., number of motor units recruited and motor unit recruitment rates), which improve SSC actions (Duchateau and Amiridis, 2023; Markovic and Mikulic, 2010). In this regard, since all the measurements in the meta-analysis studies were made via contact platforms that only measure time functioning as a stopwatch to obtain flight time, we cannot rule out that SJ executions had slight countermovement given the difficulty of performing a purely concentric jump, which is unnatural in sports actions. In this sense, it is important that future research that wishes to use SJ in their evaluations use technology such as force platforms to detect any minimal countermovement that may overestimate the test results (Merrigan et al., 2024).

### Drop jump

The meta-analysis for DJ reported a significant improvement in favor of PJT. This finding is similar to that reported by Stojanović et al. (2017) in a meta-analysis with two PJT studies in female soccer players reporting a very large effect (ES = 3.59; 95% CI: 3.04–10.23). This finding is in agreement with that reported by Sanchez et al. (2022) in a meta-analysis with six PJT studies in female soccer players, who reported significant improvements in DJ scores (ES = 0.79; *p* = 0.021). The improvements in DJ can be attributed to plyometric exercises that increase the efficacy of SSC by increasing the loading of muscles and tendons during the braking phase while aiming to decrease the duration of the transition between the braking and propulsion phases (i.e., coupling time) (Duchateau and Amiridis, 2023). These improvements are associated with neuromuscular factors such as increased recruitment and activation of high-threshold motor units and mechanical factors such as musculotendinous stiffness, which play important roles in force transmission by exerting spring-like behavior that influences subsequent muscle performance (Suchomel et al., 2018). Specifically, adaptations of tendon stiffness and structures within the muscle (i.e., actin, myosin and titin) may improve the applied force (i.e., rate of force development and power) (Suchomel et al., 2018). This, in turn, may lead to improvements in explosive actions important in soccer, such as COD, sprinting capabilities and jumping in contests with the ball or scoring goals (Suchomel et al., 2018).

### Peak power jump

Our meta-analysis for peak power jump included three studies that identified significant improvements in favor of PJT (ES = 0.85; *p* = 0.000). This finding is in line with research in female soccer players that has supported the use of PJT to improve lower limb muscle power (Bedoya et al., 2015; Slimani et al., 2016). Peak power is one of the most important variables in sports performance (Suchomel et al., 2018) and is related to key performance indicators in soccer, such as sprinting (Weyand et al., 2000), jumping (Cormie et al., 2010; McLellan et al., 2011) and COD (Suchomel et al., 2018). Even differences in peak power performance have been reported between the competitive level of starting (Baker, 2001) and substitute athletes (Barker et al., 1993; Young et al., 2005). As mentioned in the previous sections, improvements through PJT are associated with neuromuscular and mechanical factors. In this context, the mechanism thought to drive the SSC-induced improvement in maximal power is the storage and reutilization of accumulated elastic energy (Cormie et al., 2011). When a muscle-tendon unit (MTU) is stretched, mechanical work is absorbed by the MTU and may be partly stored as potential energy in the series elastic component (i.e., fiber cross-bridges, aponeurosis and tendon) (Anderson and Pandy, 1993; Cavagna and Citterio, 1974). Consequently, the area under the force‒length curve is greater during SSC (Duchateau and Amiridis, 2023), where the energy stored in the series of elastic components of the MTU is reused as a spring in the subsequent shortening contraction, generating a greater maximal power output (Cavagna and Citterio, 1974; Duchateau and Amiridis, 2023).

### Agility

Four studies were part of our meta-analysis for the Illinois test, where we identified significant improvements in favor of PJT (ES = 1.15; *p* = 0.000). This finding is similar to that reported by Ramirez-Campillo et al. (2020), who, in a meta-analysis with 5 PJT studies in female soccer players, reported significant improvements in COD speed in favor of PJT (ES = 0.73; *p* = 0.001). This finding is in agreement with the findings of Pardos-Mainer et al. (2021) in a meta-analysis with three PJT interventions in female soccer players, who reported significant improvements in favor of PJT in terms of COD speed (ES: −1.08; *p* = 0.03). Importantly, however, this meta-analysis used different tests to assess COD speed. For example, Ramirez-Campillo et al. (2020) analyzed articles that used the T-test, Meylan test, and Illinois test. In the meta-analysis of Pardos-Mainer et al. (2021), the analyzed articles used the V-cut test, and Illinois test. In contrast, our systematic review with meta-analysis included only interventions that applied the Illinois test. COD tests can differ in distance (i.e., meters) and angles of COD; for example, the Illinois test requires a much longer distance than the T-test does, whereas the T-test requires a more demanding COD (i.e., 75°) (Spiteri et al., 2014). During PJT interventions, soccer players perform exercises that require short contact times with a rapid application of force against the ground. In this context, the neuromechanical adaptations promoted by PJT can favor greater absorption of forces and increase force production per unit of time, improving the COD (Sanchez et al., 2022). In this sense, female soccer players may have developed greater braking strength through greater muscular activation of the knee flexors, which favors posterior concentric action (Sanchez et al., 2022). These improvements may offer an advantage during competitive matches, increasing the ability to COD in offensive and defensive actions (Krustruo et al., 2005).

### 20-M sprint speed

Our meta-analysis reported significant improvements in 20-m sprint speed performance for PJT (ES = 0.62; *p* = 0.01). This finding is similar to that reported in a meta-analysis by Sanchez et al. (2022) with seven articles with PJT interventions in female soccer players, who reported significant improvements in linear sprint time in favor of PJT (ES = 0.79; *p* < 0.001). This finding is similar to that reported by Pardos-Mainer et al. (2021) in a meta-analysis with five articles with PJT interventions in female soccer players, who reported significant improvements in linear sprint time in favor of PJT (ES = −1.12; *p* = 0.003). It has been suggested that sprinting is highly dependent on the ability to generate maximal force, the application of force per unit time, and the maximal power capacity of the lower limbs (Haff and Nimphius, 2012). In this sense, PJT may be favorable for inducing these improvements given its similarities with linear sprint kinematics (e.g., contact time, flight time, stride amplitude and frequency) (Alcaraz et al., 2014; Cross et al., 2014). However, to improve sprinting capacity, optimization of the force‒velocity spectrum in female soccer players may also be necessary (Leon Muñoz et al., 2024). Positive effects on linear sprint performance have been reported after strength training combined with power exercises (Suchomel et al., 2018).

### 20-M shuttle run test

No significant improvements were found for the 20-m shuttle run test in favor of the PJT (ES = 1.12; *p* = 0.13). In contrast to what was reported by Sanchez et al. (2022) in a meta-analysis with five studies with PJT in female soccer players, three studies using the 20-m shuttle run test identified significant improvements in favor of the PJT (ES = 0.60; *p* = 0.020). It has been reported that overload training methods with an emphasis on the PJT and eccentric muscle actions may be advantageous for improving neuromuscular performance, maximal strength, tendon stiffness, and force production per unit time in female soccer players (Suchomel et al., 2019). In this sense, PJT, through greater recruitment of high-threshold motor units and a more efficient SSC with a better return of the elastic energy stored during the eccentric phase of the race, could improve the performance of the 20-m shuttle run test (Turner and Jeffreys, 2010). However, for our meta-analysis, this was not the case, despite the significant improvements found for CMJ and DJ. In this context, specific sprint speed COD exercises might be necessary to induce improvements in the 20-m shuttle run test (Leon Muñoz et al., 2024). In addition to being combined with strength training through exercises involving the application of horizontal force with light, moderate and high loads can be used to enhance the specific motor actions of running (Suchomel et al., 2018).

### Kicking performance

Our meta-analysis reported significant improvements in kicking performance in ball kicking for PJT (ES = 1.30; *p* = 0.00). This finding is similar to that reported in a meta-analysis by Sanchez et al. (2022) with three studies with PJT in female soccer players, who reported a very large improvement in favor of PJT (ES = 2.24; *p* = 0.037). Kicking execution in soccer involves a high angular velocity of the hip generated through an SSC of the lower limbs and linear velocity of the foot in combination with the force applied to the ball (Rađa et al., 2019). In this sense, PJT may have generated neuromechanical adaptations such as a greater efficiency of the SSC during ball kicking with greater force production per unit of time (Suchomel et al., 2018). Although both meta-analysis demonstrated that PJT produced significant changes in kicking performance, it is important to highlight that not all studies evaluated kicking performance in the same way. For example, Ramirez-Campillo et al. (2018a) and Sanchez et al. (2022) used a radar gun. Rubley et al. (2011) measured the distance in meters from the point where the ball was kicked to the point of initial contact with the ground, which may be less precise and highly variable depending on the experience of the evaluator. On the other hand, in our meta-analysis, Rubley et al. (2011) achieved a very large improvement (ES = 1.97), similar to that reported in Sanchez et al. (2022), with very large improvements (ES = 1.37). In this regard, this could be related to the young age of the sample in the studies; the participants were 13.4 ± 0.5 years old in Rubley et al. (2011) and 16.0 ± 2.2 years old in Sanchez et al. (2022). When their results are compared with those of an adult sample, these gains decrease (ES = 0.94 and 0.87) (Ramirez-Campillo et al., 2018b). In this sense, kicking ability in soccer could improve considerably between 13 and 16 years due to the plasticity of the neuromuscular system in the developmental years of maturation (Rađa et al., 2019; Rodríguez-Lorenzo et al., 2015). Existing research on the effects on maximum speed when ball kicking, according to age, sex, dominant limb, competitive level, playing position and variations in kicking technique, reported that between 15 and 19 years, the kicking pattern is fully achieved (i.e., with a maximum kicking speed on the ball = 80–103 km/h). Finally, the differences in the mentioned studies could also be related to the volume of jumps performed during the interventions; for example, Rubley et al. (2011) performed 1,680 jumps, whereas Ramirez-Campillo et al. (2018a) performed 810 jumps. However, further interventions through PJT in female soccer players are needed to clarify these findings.

### Subgroup analysis by competitive level

Subgroup analysis by competitive level revealed significant differences in CMJ in professional female soccer players in favor of PJT (ES = 0.54; *p* = 0.03). However, no significant differences were detected in CMJ among amateur female soccer players (ES = 0.43; *p* = 0.08). Soccer is a complex team sport where performance depends on multiple factors, such as muscle strength, specifically the ability to produce force in short-term actions (e.g., jumping, COD and sprinting) (Rousopoulos et al., 2021). In this context, physical differences in vertical jump capacity have been reported between elite, subelite and recreational soccer players; these differences are significantly greater in elite soccer players (França et al., 2022; Slimani and Nikolaidis, 2019) compared to non-professional soccer players. For instance, the elite group demonstrated approximately 17% better performance in their CMJ, with an average improvement of 7 cm, and 16% better performance in the SJ with an average improvements of 6 cm, than the non-elite group (França et al., 2022; Slimani and Nikolaidis, 2019). In this sense, given the demanding explosive actions in elite soccer, it is not uncommon for these players to possess improved levels of explosive strength compared with their amateur or recreational peers (Rousopoulos et al., 2021). This could be attributed to their greater experience in sports practice, where soccer players can develop a better long-term strength baseline. However, research identifying differences in physical performance between professional and amateur female soccer players is limited. On the other hand, given the physical demands in professional soccer, sports clubs constantly seek to improve the physical performance of soccer players through their multidisciplinary teams (França et al., 2022). Such findings could explain why professional soccer players outperform amateur players in muscle strength. An important consideration is that certain training and performance characteristics, such as plyometric ability, can be influenced by muscle strength levels (Suchomel et al., 2018). Although muscle strength values ​​have not been reported in meta-analysis, it has been reported that athletes who possess greater relative strength can generate greater adaptations due to their greater neuromuscular responses, including their ability to produce higher power levels, which could explain the significant differences in CMJ after PJT for professional soccer players in our meta-analysis (Suchomel et al., 2018).

### Subgroup analysis by total number of sessions

Significant differences were found in CMJ among interventions with fewer than 16 sessions in total (ES = 0.52; *p* = 0.003). However, no significant differences were observed in CMJ between training programs with more than 16 sessions (ES = 1.06; *p* = 0.29). Unlike what was reported by Ramirez-Campillo et al. (2020) in a meta-analysis that aimed to identify the effects of PJT on vertical jump height in female soccer players, they reported moderate effects on vertical jump height in the subgroup analysis of the total number of PJT sessions (i.e., <12 sessions and >12 sessions), with no significant differences between subgroups (*p* = 0.96). Our findings suggest that a greater number of PJT sessions induces greater gains in vertical jump height when a greater number of jumps is performed. However, our subgroup analysis contrasts with this theory. For example, the two studies with the longest training sessions (i.e., 36 sessions) had a total volume of 3,240 jumps (Campo et al., 2009; Fischetti et al., 2019), with a volume of 90 jumps per session. The studies with the shortest training sessions (i.e., eight sessions) had a total volume of 640 jumps (Sanchez et al., 2022) and 524 jumps (Maciejczyk et al., 2021), equivalent to 80 jumps per session (Sanchez et al., 2022) and 65 jumps per session (Sanchez et al., 2022). On the other hand, studies with <16 training sessions were characterized by the inclusion of bipodal and unipodal plyometric exercises in multiple directions compared with studies with >16 training sessions. For example, programs with fewer than 16 training sessions included unilateral, bilateral, cyclic (i.e., repeated), acyclic (i.e., non-repeated), vertical, horizontal, lateral, and turning jumps (Leon Muñoz et al., 2024; Maciejczyk et al., 2021; Ramirez-Campillo et al., 2018a; Rosas et al., 2017; Sanchez et al., 2022). These were characterized by short (i.e., <250 ms foot-ground contact time) and slow (i.e., ≥250 ms foot-ground contact time) SSCs. Furthermore, Ramirez-Campillo et al. (2018b) & Rosas et al. (2017) used individualized box heights (i.e., 5–35 cm) during DJs, which were calculated through the reactive strength index. In contrast, the jumps used by studies with >16 training sessions were characterized only by the execution of vertical and horizontal bipodal jumps (Campo et al., 2009; Fischetti et al., 2019; Porrati-Paladino and Cuesta-Barriuso, 2021; Ramírez-Campillo et al., 2016). This finding is relevant, given that the literature has suggested that the combination of unilateral and bilateral exercises seems more advantageous for inducing superior improvements in performance (Cao et al., 2024; Ramírez-Campillo et al., 2015). Therefore, the characteristics of the PJT interventions, as well as the competitive level of the players in the analyzed studies, may help explain our findings.

### Subgroup analysis by training duration

CMJ did not significantly differ across studies lasting less than 6 weeks (ES = 0.14; *p* = 0.60). In contrast, significant differences were observed in CMJ in studies lasting longer than 6 weeks (ES = 1.42; *p* = 0.05). This finding is similar to that reported in a meta-analysis by Ramirez-Campillo et al. (2020) on the effects of PJT on vertical jump height in female soccer players, who reported that interventions lasting 8 weeks or longer had a greater effect (ES = 1.24) than did interventions lasting less than 8 weeks, with a moderate effect (ES = 0.66). This finding is in line with that reported by Stojanović et al. (2017) in a meta-analysis on the effects of PJT on vertical jump height in female athletes, who reported that interventions lasting longer than 10 weeks had greater effects on female athletes (ES = 1.87). In this context, a longer duration of PJT interventions could facilitate neuromechanical adaptations associated with increased vertical jump (Ramirez-Campillo et al., 2020). Indeed, it has been suggested that more than 8 weeks of systematic application of PJT are needed to improve muscle strength manifestations in elite team sport athletes (Slimani and Nikolaidis, 2019). In addition, PJT interventions with a longer duration may allow the accumulation of a greater volume of jumps, which could lead to greater improvements in vertical jump height (Asadi et al., 2018; de Villarreal et al., 2009). However, current evidence suggests that key moderating variables of PJT, such as volume, intensity, type of exercise, type of surface, and training level, should be considered to maximize its effects on physical performance (Ramirez-Campillo et al., 2018a).

### Subgroup analysis by frequency of training

No significant differences were observed in CMJ with training frequencies greater than two sessions per week (ES = 0.95; *p* = 0.19). However, significant differences were found in CMJ in groups training fewer than two sessions per week (ES = 0.50; *p* = 0.005). This finding is similar to that reported by Ramirez-Campillo et al. (2020) in a meta-analysis on the effects of PJT on vertical jump height in female soccer players, where they reported a moderate effect (ES = 0.80) with interventions with two or more sessions per week, whereas interventions with fewer than two sessions per week produced a large effect (ES = 1.47). In contrast, the meta-analysis by Ramirez-Campillo et al. (2020) included only three studies for the analysis of the effects of fewer than two sessions per week. Our meta-analysis included seven studies and eight experimental groups, which reinforces the aforementioned findings. On the other hand, one of the studies included in our meta-analysis, Ramirez-Campillo et al. (2018b) compared the effects of 1 vs. 2 PJT sessions per week; matched for total volume, intensity, and jumping exercises; and reported similar gains in vertical jump height. Similar to that reported by Hernandez-Martinez et al. (2023) in male volleyball players, a single session of PJT per week was as effective as two sessions per week in achieving significant improvements in vertical jump performance. Similarly, Bouguezzi et al. (2020) reported that when performing a moderate volume of jumping (e.g., 680 jumps), an increased frequency of the PTJ over 8 weeks had no additional effects on athletic performance measures in prepubertal male soccer players. Practically, the use of a lower PJT frequency may help female players dedicate more time to other relevant physical capacities in their preparation, which could help to save hours of training and optimize their sport performance (Bouguezzi et al., 2020). Finally, current evidence suggests that it is important to consider the moderating role of PJT frequency in optimizing vertical jump height gains in female soccer players. To do so, it is relevant to consider key PJT moderating variables such as volume, intensity, and frequency.

### Practical applications

The lack of PJT research in female soccer players has been reported previously (Ramirez-Campillo et al., 2018a). However, our systematic review with meta-analysis presented 14 RCTs, which is a larger number of studies than previous meta-analysis (Ramirez-Campillo et al., 2020). On the basis of the results of our meta-analysis, we suggest several practical applications:

• Female soccer players can incorporate PJT programs into their regular training to improve vertical jumps (CMJ, SJ and DJ), Illinois tests, 20-m shuttle runs, 20-m speeds, and ball kicking tests.
• PJT can be effective for both young and adult female soccer players (age range: 15–26 years), with or without previous PJT experience, from amateur to professional level.
• Compared with their amateur peers, professional female soccer players might present greater improvements in CMJ height.
• A training frequency of 1–2 sessions per week for 6–12 weeks, with a maximal linear-maximal intensity, may be an adequate stimulus to improve physical performance.
• The combination of single-leg and two-leg jumps with the application of vertical, horizontal and lateral forces could be more effective in increasing the height of the vertical jump in female soccer players.

### Limitations and strengths

Our systematic review with meta-analysis was not free of limitations: (i) more than half of the studies had a high risk of bias (57%); (ii) it was not possible to use chronological age as a moderator variable since only two studies included young athletes; and (iii) the studies analyzed did not report on menstrual cycle control in the participants. On the other hand, the strengths of our meta-analysis include: (i) a methodological quality above 60% in the studies analyzed; (ii) the use of methodological processes governed by PRISMA, PROSPERO, TESTEX, RoB 2 and GRADE; (iii) the use of six generic databases: PubMed, Medline, Psychology and Behavioral Sciences Collection (EBSCO), CINAHL Complete, Scopus and Web of Science; and (iv) the execution of a meta-analysis by subgroups across the competitive level, total number of sessions, duration of the intervention and frequency of training.

## Conclusion

PJT leads to significant improvements in vertical jump (CMJ, SJ, DJ and peak power jump), Illinois, 20-m speed, and ball kicking in female soccer players. With respect to the competitive level, only amateur players show significant improvements in CMJ, whereas total sessions ≤16 sessions and a frequency <2 sessions per week lead to improvements in CMJ. Therefore, PJT is an effective and economical method that improves physical performance in female soccer players. However, PJT is not a predictor of physical performance (duration, frequency or number of sessions).

## Data Availability

The original contributions presented in the study are included in the article/Supplementary Material, further inquiries can be directed to the corresponding author.
