# Supplementary material for: Effects of plyometric jump training on physical performance in female soccer players across the competitive level: a systematic review with meta-analysis of randomized controlled trials
Source: Front Physiol. 2025 Oct 1;16:1675849. doi: 10.3389/fphys.2025.1675849 (PMC12521099; doi:10.3389/fphys.2025.1675849)
Supplement: Supplementary file 1 [file DataSheet1.pdf]

Supplementary Material

Vertical Jump

Study name	Hedges's g	Standard error	Variance	Lower limit	Upper limit	Z-Value	p-Value
Porrati et al. (2021)	0,033	0,487	0,237	-0,921	0,988	0,069	0,945
Ramírez-Campillo et al. (2018) a	0,565	0,631	0,398	-0,671	1,801	0,896	0,370
Ramírez-Campillo et al. (2015)	-1,135	0,343	0,118	-1,808	-0,462	-3,306	0,001
Campo et al. (2009)	9,005	1,487	2,211	6,091	11,919	6,056	0,000
Leon et al. (2024)	-0,000	0,402	0,162	-0,789	0,789	-0,000	1,000
Fischetti et al. (2019)	-0,945	0,388	0,151	-1,706	-0,185	-2,436	0,015
Maciejczyk et al. (2021)	0,633	0,501	0,251	-0,348	1,615	1,265	0,206
Sanches et al. (2022)	0,881	0,532	0,283	-0,162	1,924	1,655	0,098
Moulay et al. (2022)	1,387	0,460	0,212	0,485	2,289	3,013	0,003
Rosas et al. (2017)	0,464	0,468	0,219	-0,453	1,382	0,992	0,321
Ramírez-Campillo et al. (2016)	0,514	0,436	0,190	-0,340	1,369	1,180	0,238
Nonnato et al. (2020)	0,834	0,495	0,245	-0,137	1,804	1,684	0,092
Ramírez-Campillo et al. (2018) b	0,700	0,583	0,340	-0,443	1,843	1,200	0,230
	0,591	0,333	0,111	-0,061	1,243	1,776	0,076

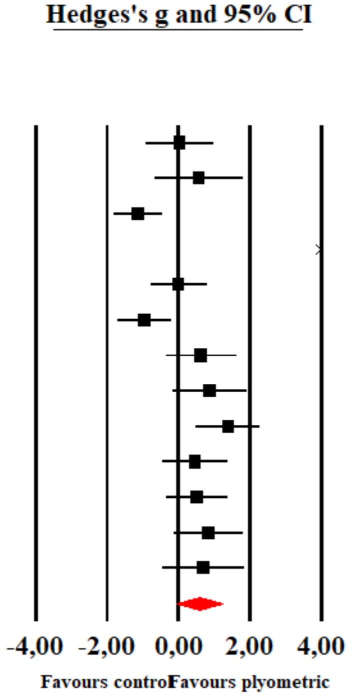

Figure S1. Forest plot of changes in CMJ in female soccer players participating in plyometric training compared with female soccer players assigned as controls. Values shown are effect sizes (Hedges' g) with 95% confidence intervals (CI). The size of the squares plotted reflects the statistical weight of each study.

Study name	Hedges's g	Standard error	Variance	Lower limit	Upper limit	Z-Value	p-Value
Nonnato et al. (2022)	0,066	0,473	0,224	-0,861	0,993	0,140	0,889
Maciejczyk et al. (2021)	0,588	0,342	0,117	-0,083	1,259	1,718	0,086
Moulay idriss et al. (2022)	1,377	0,460	0,211	0,476	2,279	2,996	0,003
Rosas et al. (2017)	0,748	0,479	0,229	-0,190	1,686	1,563	0,118
Ramírez-Campillo et al. (2016)	1,095	0,462	0,213	0,189	2,000	2,369	0,018
	0,760	0,212	0,045	0,345	1,175	3,589	0,000

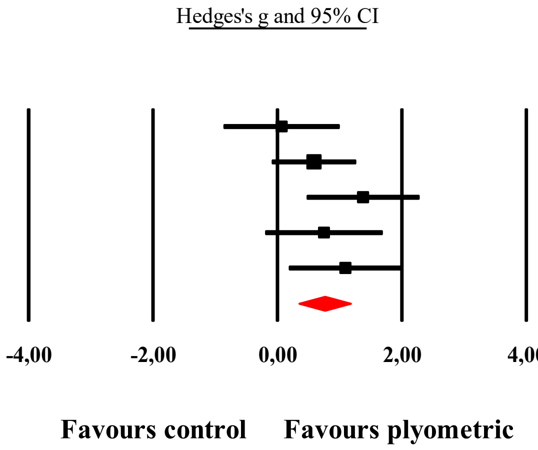

Figure S2. Forest plot of changes in SJ in female soccer players participating in plyometric training compared with female soccer players assigned as controls. Values shown are effect sizes (Hedges' g) with 95% confidence intervals (CI). The size of the squares plotted reflects the statistical weight of each study.

Study name	Statistics for each study						
	Hedges's g	Standard error	Variance	Lower limit	Upper limit	Z-Value	p-Value
Campo et al. (2009)	5,433	0,960	0,921	3,552	7,315	5,660	0,000
Ramírez-Campillo et al. (2018) a	0,897	0,594	0,353	-0,268	2,061	1,509	0,131
Ramírez-Campillo et al. (2018) b	1,012	0,655	0,430	-0,273	2,296	1,543	0,123
Ramírez-Campillo et al. (2015)	1,772	0,377	0,142	1,033	2,511	4,699	0,000
Rosas et al. (2017)	1,458	0,525	0,275	0,429	2,486	2,779	0,005
Ramírez-Campillo et al. (2016)	0,527	0,436	0,190	-0,329	1,382	1,207	0,228
	1,653	0,487	0,237	0,699	2,607	3,397	0,001

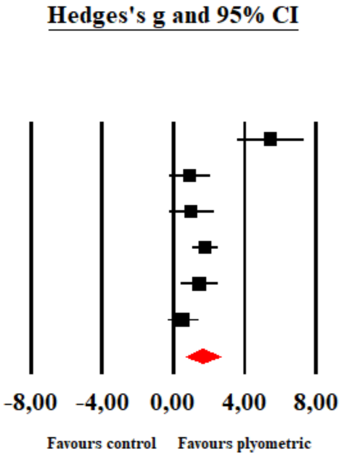

Figure S3. Forest plot of changes in DJ in female soccer players participating in plyometric training compared with female soccer players assigned as controls. Values shown are effect sizes (Hedges' g) with 95% confidence intervals (CI). The size of the squares plotted reflects the statistical weight of each study.

Study name	Statistics for each study						
	Hedges's g	Standard error	Variance	Lower limit	Upper limit	Z-Value	p-Value
Ozbar et al. (2014)	0,96	0,48	0,23	0,03	1,90	2,02	0,04
Ramírez-Campillo et al. (2016)	0,47	0,43	0,19	-0,38	1,32	1,08	0,28
Rosas et al. (2017)	1,26	0,51	0,26	0,26	2,25	2,47	0,01
	0,85	0,27	0,07	0,32	1,38	3,14	0,00

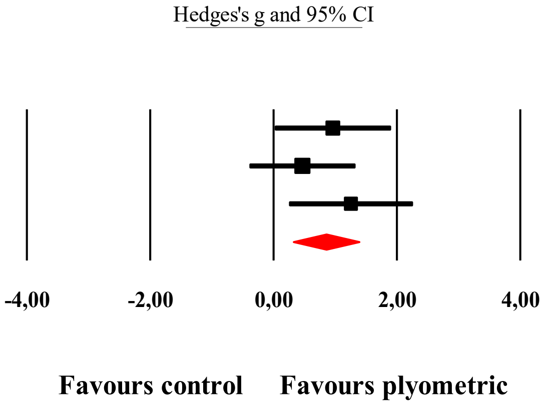

Figure S4. Forest plot of changes in Peak Power Jump in female soccer players participating in plyometric training compared with female soccer players assigned as controls. Values shown are effect sizes (Hedges' g) with 95% confidence intervals (CI). The size of the squares plotted reflects the statistical weight of each study.

Agility

Study name	Hedges's g	Standard error	Variance	Lower limit	Upper limit	Z-Value	p-Value
Maciejczyk et al. (2021)	0,93	0,52	0,27	-0,08	1,94	1,80	0,07
Ramírez-Campillo et al. (2016)	1,24	0,35	0,12	0,55	1,92	3,55	0,00
Rosas et al. (2017)	0,00	0,46	0,21	-0,90	0,90	0,00	1,00
Ramírez-Campillo et al. (2015)	2,00	0,39	0,15	1,23	2,77	5,10	0,00
	1,15	0,21	0,04	0,74	1,56	5,54	0,00

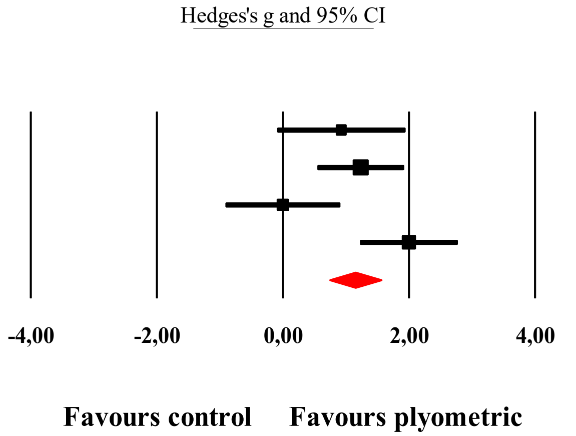

Figure S5. Forest plot of changes in Illinois test in female soccer players participating in plyometric training compared with female soccer players assigned as controls. Values shown are effect sizes (Hedges' g) with 95% confidence intervals (CI). The size of the squares plotted reflects the statistical weight of each study.

Study name	Hedges's g	Standard error	Variance	Lower limit	Upper limit	Z-Value	p-Value
Ramírez-Campillo et al. (2015)	0,78	0,33	0,11	0,13	1,43	2,36	0,02
Ramírez-Campillo et al. (2016)	0,43	0,43	0,19	-0,42	1,28	0,99	0,32
Rosas et al. (2017)	0,53	0,47	0,22	-0,39	1,45	1,13	0,26
	0,62	0,23	0,05	0,17	1,07	2,71	0,01

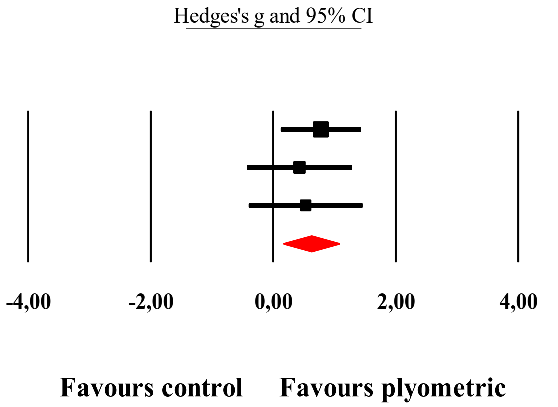

Figure S6. Forest plot of changes in 20-meters speed in female soccer players participating in plyometric jump training compared with female soccer players assigned as controls. Values shown are effect sizes (Hedges; g) with 95% confidence intervals (CI). The size of the squares plotted reflects the statistical weight of each study.

Ball Kicking performance

Study name	Hedges's g	Standard error	Variance	Lower limit	Upper limit	Z-Value	p-Value
Sanches et al. (2022)	1,375	0,568	0,323	0,261	2,489	2,419	0,016
Ramirez-Campillo et al. (2018) a	0,946	0,597	0,357	-0,225	2,117	1,583	0,113
Rubley et al. (2011)	1,978	0,600	0,361	0,801	3,155	3,293	0,001
Ramirez-Campillo et al. (2018) b	0,870	0,646	0,418	-0,397	2,136	1,346	0,178
	1,308	0,301	0,090	0,719	1,897	4,351	0,000

(Statistics for each study)

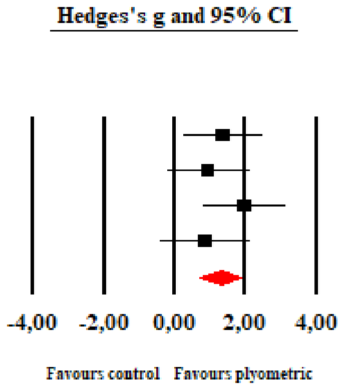

Figure S7. Forest plot of changes in ball kicking both foot in female soccer players participating in plyometric training compared with female soccer players assigned as controls. Values shown are effect sizes (Hedges' g) with 95% confidence intervals (CI). The size of the squares plotted reflects the statistical weight of each study.

Sprint performance

Study name	Hedges's g	Standard error	Variance	Lower limit	Upper limit	Z-Value	p-Value
Leon et al. (2024)	2,552	0,551	0,303	1,472	3,631	4,632	0,000
Sanches et al. (2022)	-0,914	0,534	0,285	-1,961	0,133	-1,710	0,087
Rosas et al. (2017)	0,887	0,486	0,236	-0,065	1,839	1,827	0,068
Ozbar et al. (2014)	2,008	0,560	0,314	0,910	3,105	3,586	0,000
	1,126	0,751	0,564	-0,346	2,599	1,499	0,134

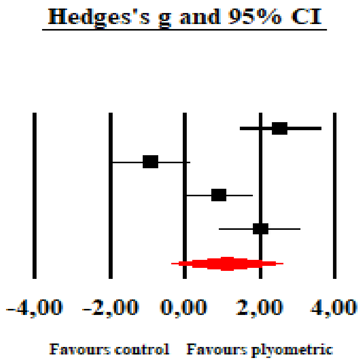

Figure S8. Forest plot of changes in 20-meters speed in female soccer players participating in plyometric training compared with female soccer players assigned as controls. Values shown are effect sizes (Hedges' g) with 95% confidence intervals (CI). The size of the squares plotted reflects the statistical weight of each study.

Meta-analysis sub group

Study name	Subgroup within study	Hedges's g	Standard error	Variance	Lower limit	Upper limit	Z-Value	p-Value
		Statistics for each study						
Ramirez-Campillo et al. (2018) a	A	0,565	0,631	0,398	-0,671	1,801	0,896	0,370
Ramirez-Campillo et al. (2016)	A	0,514	0,436	0,190	-0,340	1,369	1,180	0,238
Ramirez-Campillo et al. (2018) b	A	0,700	0,583	0,340	-0,443	1,843	1,200	0,230
Rosas et al. (2017)	A	0,464	0,468	0,219	-0,453	1,382	0,992	0,321
		0,543	0,256	0,065	0,042	1,045	2,125	0,034
Fischetti et al. (2019)	B	-0,945	0,388	0,151	-1,706	-0,185	-2,436	0,015
Leon et al. (2024)	B	-0,000	0,402	0,162	-0,789	0,789	-0,000	1,000
Maciejczyk et al. (2021)	B	0,633	0,501	0,251	-0,348	1,615	1,265	0,206
Moulay et al. (2022)	B	1,387	0,460	0,212	0,485	2,289	3,013	0,003
Sanches et al. (2022)	B	0,881	0,532	0,283	-0,162	1,924	1,655	0,098
Nonnato et al. (2020)	B	0,834	0,495	0,245	-0,137	1,804	1,684	0,092
		0,431	0,367	0,134	-0,288	1,150	1,175	0,240

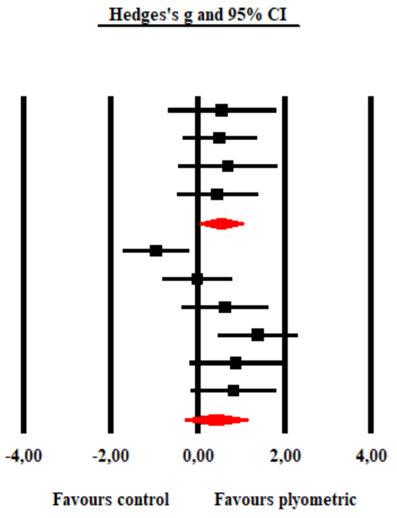

Figure S9. Forest plot of changes in CMJ in female soccer players participating in plyometric training compared with female soccer players assigned as controls, according to competitive level: A= amateur; B= professional. Values shown are effect sizes (Hedges' g) with 95% confidence intervals (CI). The size of the squares plotted reflects the statistical weight of each study.

Study name	Subgroup within study	Hedges's g	Standard error	Variance	Lower limit	Upper limit	Z-Value	p-Value
		Statistics for each study						
Porrati et al. (2021)	6 weeks or <	0,033	0,487	0,237	-0,921	0,988	0,069	0,945
Ramirez-Campillo et al. (2015)	6 weeks or <	-1,135	0,343	0,118	-1,808	-0,462	-3,306	0,001
Leon et al. (2024)	6 weeks or <	-0,000	0,402	0,162	-0,789	0,789	-0,000	1,000
Maciejczyk et al. (2021)	6 weeks or <	0,633	0,501	0,251	-0,348	1,615	1,265	0,206
Sanches et al. (2022)	6 weeks or <	0,881	0,532	0,283	-0,162	1,924	1,655	0,098
Rosas et al. (2017)	6 weeks or <	0,464	0,468	0,219	-0,453	1,382	0,992	0,321
Ramirez-Campillo et al. (2016)	6 weeks or <	0,514	0,436	0,190	-0,340	1,369	1,180	0,238
		0,149	0,289	0,083	-0,417	0,715	0,515	0,606
Ramirez-Campillo et al. (2018) a	7 weeks or >	0,565	0,631	0,398	-0,671	1,801	0,896	0,370
Campo et al. (2009)	7 weeks or >	9,005	1,487	2,211	6,091	11,919	6,056	0,000
Fischetti et al. (2019)	7 weeks or >	-0,945	0,388	0,151	-1,706	-0,185	-2,436	0,015
Moulay et al. (2022)	7 weeks or >	1,387	0,460	0,212	0,485	2,289	3,013	0,003
Nonnato et al. (2020)	7 weeks or >	0,834	0,495	0,245	-0,137	1,804	1,684	0,092
Ramirez-Campillo et al. (2018) b	7 weeks or >	0,700	0,583	0,340	-0,443	1,843	1,200	0,230
		1,420	0,732	0,536	-0,015	2,855	1,939	0,052

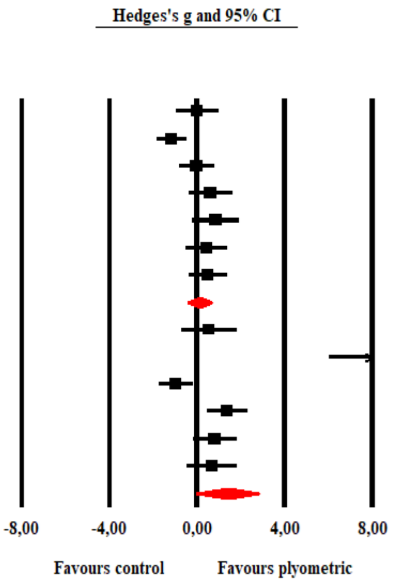

Figure S10. Forest plot of changes in CMJ in female soccer players participating in plyometric training compared with female soccer players assigned as controls, according to duration of intervention. Values shown are effect sizes (Hedges' g) with 95% confidence intervals (CI). The size of the squares plotted reflects the statistical weight of each study.

Study name	Subgroup within study	Statistics for each study						
		Hedges's g	Standard error	Variance	Lower limit	Upper limit	Z-Value	p-Value
Ramirez-Campillo et al. (2018) a	<16 total sessions	0,565	0,631	0,398	-0,671	1,801	0,896	0,370
Leon et al. (2024)	<16 total sessions	-0,000	0,402	0,162	-0,789	0,789	-0,000	1,000
Maciejczyk et al. (2021)	<16 total sessions	0,633	0,501	0,251	-0,348	1,615	1,265	0,206
Sanches et al. (2022)	<16 total sessions	0,881	0,532	0,283	-0,162	1,924	1,655	0,098
Rosas et al. (2017)	<16 total sessions	0,464	0,468	0,219	-0,453	1,382	0,992	0,321
Ramirez-Campillo et al. (2016)	<16 total sessions	0,514	0,436	0,190	-0,340	1,369	1,180	0,238
Nonnato et al. (2020)	<16 total sessions	0,834	0,495	0,245	-0,137	1,804	1,684	0,092
Ramirez-Campillo et al. (2018) b	<16 total sessions	0,700	0,583	0,340	-0,443	1,843	1,200	0,230
		0,525	0,174	0,030	0,184	0,865	3,016	0,003
Porrati et al. (2021)	>16 total sessions	0,033	0,487	0,237	-0,921	0,988	0,069	0,945
Ramirez-Campillo et al. (2015)	>16 total sessions	-1,135	0,343	0,118	-1,808	-0,462	-3,306	0,001
Campo et al. (2009)	>16 total sessions	9,005	1,487	2,211	6,091	11,919	6,056	0,000
Fischetti et al. (2019)	>16 total sessions	-0,945	0,388	0,151	-1,706	-0,185	-2,436	0,015
Moulay et al. (2022)	>16 total sessions	1,387	0,460	0,212	0,485	2,289	3,013	0,003
		1,061	0,852	0,725	-0,608	2,730	1,246	0,213

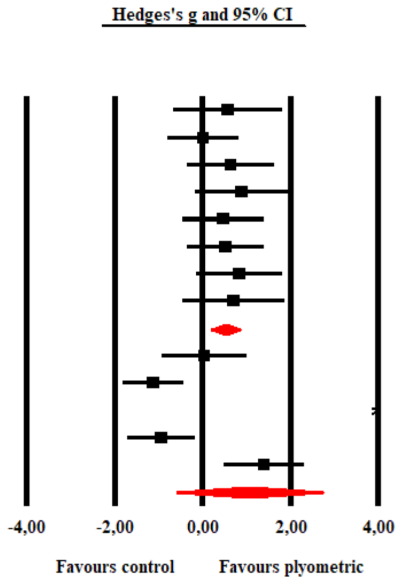

Figure S11. Forest plot of changes in CMJ in female soccer players participating in plyometric training compared with female soccer players assigned as controls, according to volume of training. Values shown are effect sizes (Hedges' g) with 95% confidence intervals (CI). The size of the squares plotted reflects the statistical weight of each study.

Study name	Subgroup within study	Statistics for each study						
		Hedges's g	Standard error	Variance	Lower limit	Upper limit	Z-Value	p-Value
Ramirez-Campillo et al. (2018) a	2 sessions or <	0,565	0,631	0,398	-0,671	1,801	0,896	0,370
Leon et al. (2024)	2 sessions or <	-0,000	0,402	0,162	-0,789	0,789	-0,000	1,000
Maciejczyk et al. (2021)	2 sessions or <	0,633	0,501	0,251	-0,348	1,615	1,265	0,206
Sanches et al. (2022)	2 sessions or <	0,881	0,532	0,283	-0,162	1,924	1,655	0,098
Rosas et al. (2017)	2 sessions or <	0,464	0,468	0,219	-0,453	1,382	0,992	0,321
Ramirez-Campillo et al. (2016)	2 sessions or <	0,514	0,436	0,190	-0,340	1,369	1,180	0,238
Nonnato et al. (2020)	2 sessions or <	0,834	0,495	0,245	-0,137	1,804	1,684	0,092
Ramirez-Campillo et al. (2018) b	2 sessions or <	0,700	0,583	0,340	-0,443	1,843	1,200	0,230
		0,525	0,174	0,030	0,184	0,865	3,016	0,003
Porrati et al. (2021)	3 sessions or >	0,033	0,487	0,237	-0,921	0,988	0,069	0,945
Ramirez-Campillo et al. (2015)	3 sessions or >	-1,135	0,343	0,118	-1,808	-0,462	-3,306	0,001
Campo et al. (2009)	3 sessions or >	9,005	1,487	2,211	6,091	11,919	6,056	0,000
Fischetti et al. (2019)	3 sessions or >	-0,945	0,388	0,151	-1,706	-0,185	-2,436	0,015
Moulay et al. (2022)	3 sessions or >	1,387	0,460	0,212	0,485	2,289	3,013	0,003
		1,061	0,852	0,725	-0,608	2,730	1,246	0,213

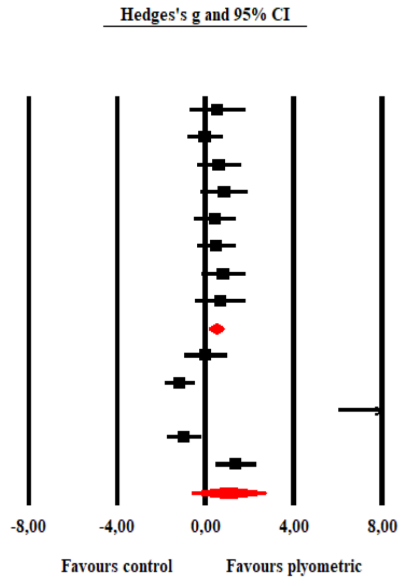

Figure S12. Forest plot of changes in CMJ in female soccer players participating in plyometric training compared with female soccer players assigned as controls, according to frequency of training. Values shown are effect sizes (Hedges' g) with 95% confidence intervals (CI). The size of the squares plotted reflects the statistical weight of each study.
